# Expression of L1 retrotransposons in granulocytes from patients with active systemic lupus erythematosus

**DOI:** 10.1186/s13100-023-00293-7

**Published:** 2023-05-10

**Authors:** Kennedy C. Ukadike, Rayan Najjar, Kathryn Ni, Amanda Laine, Xiaoxing Wang, Alison Bays, Martin S. Taylor, John LaCava, Tomas Mustelin

**Affiliations:** 1 Department of Medicine, Division of Rheumatology, University of Washington, 750 Republican Street, Room E507, Seattle, WA 99108 USA; 2 Department of Internal Medicine, Renown Rheumatology, Renown Health – University of Nevada, Reno School of Medicine, 75 Pringle Way, Suite 701, Reno, NV 89502 USA; 3 Department of Pathology, Massachusetts General Hospital, Boston, MA USA; 4 Laboratory of Cellular and Structural Biology, The Rockefeller University, New York, NY USA; 5 European Research Institute for the Biology of Ageing, University Medical Center Groningen, Groningen, The Netherlands

**Keywords:** L1, Retrotransposon, Systemic lupus erythematosus, Neutrophils, Interferon

## Abstract

**Background:**

Patients with systemic lupus erythematosus (SLE) have autoantibodies against the L1-encoded open-reading frame 1 protein (ORF1p). Here, we report (i) which immune cells ORF1p emanates from, (ii) which L1 loci are transcriptionally active, (iii) whether the cells express L1-dependent interferon and interferon-stimulated genes, and (iv) the effect of inhibition of L1 ORF2p by reverse transcriptase inhibitors.

**Results:**

L1 ORF1p was detected by flow cytometry primarily in SLE CD66b^+^CD15^+^ regular and low-density granulocytes, but much less in other immune cell lineages. The amount of ORF1p was higher in neutrophils from patients with SLE disease activity index (SLEDAI) > 6 (*p* = 0.011) compared to patients with inactive disease, SLEDAI < 4. Patient neutrophils transcribed seven to twelve human-specific L1 loci (L1Hs), but only 3 that are full-length and with an intact ORF1. Besides serving as a source of detectable ORF1p, the most abundant transcript encoded a truncated ORF2p reverse transcriptase predicted to remain cytosolic, while the two other encoded an intact full-length ORF2p. A number of genes encoding proteins that influence L1 transcription positively or negatively were altered in patients, particularly those with active disease, compared to healthy controls. Components of nucleic acid sensing and interferon induction were also altered. SLE neutrophils also expressed type I interferon-inducible genes and interferon β, which were substantially reduced after treatment of the cells with drugs known to inhibit ORF2p reverse transcriptase activity.

**Conclusions:**

We identified L1Hs loci that are transcriptionally active in SLE neutrophils, and a reduction in the epigenetic silencing mechanisms that normally counteract L1 transcription. SLE neutrophils contained L1-encoded ORF1p protein, as well as activation of the type I interferon system, which was inhibited by treatment with reverse transcriptase inhibitors. Our findings will enable a deeper analysis of L1 dysregulation and its potential role in SLE pathogenesis.

**Supplementary Information:**

The online version contains supplementary material available at 10.1186/s13100-023-00293-7.

## Background

Autoantibodies against nucleic acids and proteins associated with them, as well as elevated type I interferons in 60–90% of patients [1–3] are hallmarks of systemic lupus erythematosus (SLE), a systemic and often severe autoimmune disease. We recently reported that a majority of adult [4] and pediatric [5] SLE patients also have autoantibodies against one of the two proteins encoded by the long interspersed element-1 (LINE-1 or L1), termed ORF1p. These autoantibodies are higher in patients with active disease than in those in remission and they correlate positively with many measures of disease activity, such as the SLE disease activity index (SLEDAI), complement consumption, anti-double-stranded DNA antibodies, other autoantibodies, type I interferons, and the presence of lupus nephritis [4, 5].

The L1 retrotransposon has been extraordinarily prolific over evolutionary time: it is present in the genomes of organisms in all kingdoms of life, often in large copy numbers. There are over 500,000 copies of L1 in our own genome, constituting approximately 17% of it [6–8]. L1 sequences are present both within introns of protein-coding genes and in intergenic regions. The vast majority of these L1 sequences are incomplete (most often 5’ truncated) and contain numerous mutations that disrupt the two open reading-frames, termed *ORF1* and *ORF2*; these loci cannot produce the encoded proteins or use them to retrotranspose. Only 90 L1 have intact ORFs and 82 are full-length and seemingly intact, but as few as six of them are still ‘hot’[9], *i.e.,* these loci are mobile in contemporary humans.

While there is currently no indication that de novo insertions of L1 play any role in SLE or other autoimmune diseases, the L1-encoded proteins have the capacity to cause pathology by other mechanisms [10]. The protein encoded by *ORF2*, termed ORF2p, is an active reverse transcriptase [11, 12], which uses RNA as a template to synthesize a DNA copy of the RNA sequence. The first product is an RNA:DNA heteroduplex; in the canonical L1 lifecycle, this occurs within the nucleus as part of a mechanism termed target-primed reverse transcription [13]. Unexpectedly, both RNA:DNA heteroduplexes and single-stranded DNA have been detected in the cytosol in cells with active L1 [14, 15], indicating that cytosolic reverse transcription also occurs. Furthermore, the synthesized DNA can trigger DNA sensors, such as cyclic-guanine adenosine synthase (cGAS)[16], ZCCHC3 [17, 18], and ZBP1 [19, 20], the physiological functions of which are to detect cytosolic pathogen-derived DNA. It was recently reported [21] that L1 ORF2p-catalyzed reverse transcription, cGAS-mediated DNA-sensing, and the resulting IFNβ production operate during late cellular senescence both in cell culture and in intact mice and, presumably, in humans. The produced IFNβ contributed to the inflammation associated with aging, but was eliminated by reverse transcriptase inhibitors [21].

The polypeptide encoded by *ORF1*, termed ORF1p, is ~ 40 kDa in mass and forms homotrimeric [22, 23] and oligomeric [24] proteins with RNA-binding, nucleic-acid-chaperone properties that coat the L1 RNA; ORF1p is necessary for L1 retrotransposition (reviewed in [25, 26]). ORF1p:ORF2p interactions apparently depend on the presence of intact RNA [18, 27]. Importantly, under ectopic expression in HEK-293 T cells, ORF1p is present in up to ~ tens of copies per ORF2p in affinity-enriched L1 ribonucleoprotein (RNP) [27], but in endogenously expressing cell lines and human cancer tissues [28], the stoichiometry of ORF1p:ORF2p is much higher. Endogenous ORF1p has been shown to accumulate in the cytoplasm [29] and to populate RNA (stress) granules [30]. Studies using ectopic L1 expression have also provided evidence that L1 RNP accumulates within stress granules [30–34]), although alternative characterizations have also been proposed [18, 35]. ORF1p- and L1-related RNPs co-assemble with a diverse range of nucleic acids [18, 35, 36]; the L1 RNA itself exhibits immunogenic viral mimicry [36]. ORF1p- and L1-related RNPs associate with several well-known autoantigens in SLE, such as RO60 [27, 37–39]. SLE patients also have autoantibodies that recognize several of the other cellular proteins that associate with ORF1p in the RNA-rich macromolecular aggregates (manuscript submitted). While ORF1p is not able to drive retrotransposition without the participation of ORF2p, one could envision that ORF1p by virtue of its immunogenicity could play a role in SLE by forming immune complexes with autoantibodies that recognize it [10]. Such immune complexes could be deposited in tissues, such as the kidney, and contribute to the tissue inflammation observed in patients with SLE.

The presence of autoantibodies in SLE patients that react with ORF1p suggests that this protein is, or recently was, expressed in patients. Indeed, it was detected by immunoblotting and immunohistochemistry in salivary glandular cells from a Sjögren’s syndrome patient and in a renal biopsy from a lupus nephritis patient [40, 41]. The positive correlation of autoantibodies against ORF1p with disease activity measured by SLEDAI or complement consumption also suggests that the presence of the protein may be specifically associated with active disease. To test this, we analyzed immune cells isolated from the blood of patients with SLE for the presence of ORF1p protein, L1 transcripts, L1 regulators, and reverse transcriptase-dependent IFNβ and IFN-inducible genes pre- and post-treatment with reverse transcriptase inhibitors.

## Results

### Detection of ORF1p in SLE neutrophils by flow cytometry

**Fig. 1:**
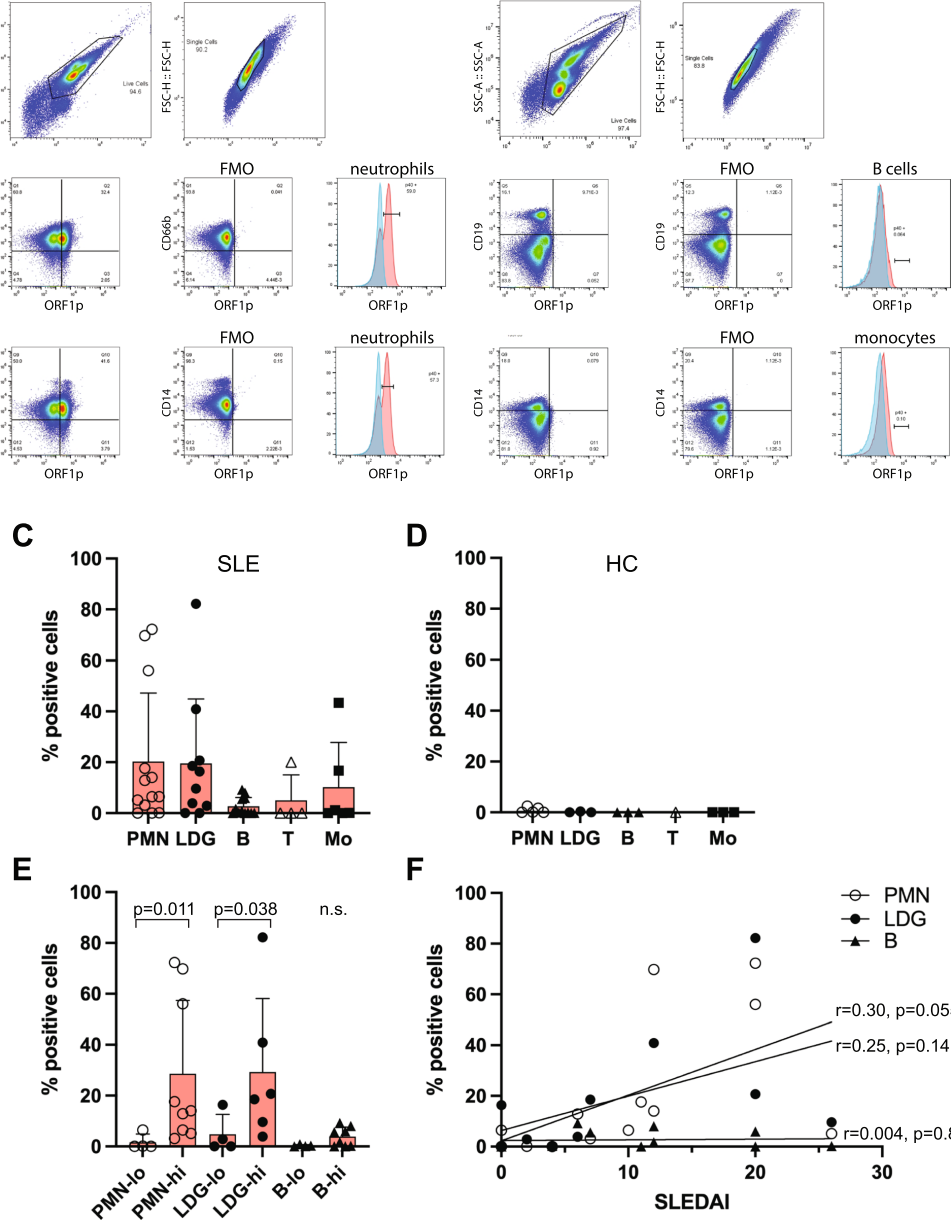
Presence of L1 ORF1p in neutrophils from SLE patients. **A** Flow cytometry of PMN from a representative SLE patient with active disease stained for ORF1p and the lineage markers CD66b (middle row) and CD14 (bottom row) compared to cells stained with all antibodies except ORF1p (FMO, fluorescence minus one). **B** Flow cytometry of PBMC from the same SLE patient stained for ORF1p and the lineage markers CD19 (middle row) and CD14 (bottom row) compared to FMO controls. **C** Summary of the percent ORF1p + leukocytes in 13 SLE patients: neutrophils (CD66b^+^ PMN), low-density granulocytes (CD66b^+^ PBMC), B cells (CD19 + PBMC), T cells (CD3^+^ PBMC), and monocytes (CD14^+^ PBMC). Error bars represent standard deviation. **D**. Summary of the percent ORF1p^+^ leukocytes in 6 healthy controls (HC) in the same cell lineages. **E** The percent ORF1p^+^ neutrophils (open circles), low-density granulocytes (filled circles), and B cells (filled triangles) in patients with low SLEDAI < 4 (*n* = 4; -lo) and patients with high SLEDAI > 6 (*n* = 9; -hi). **F** Linear regression analysis of the percentages of ORF1p^+^ neutrophils (open circles), low-density granulocytes (filled circles), and B cells (filled triangles) versus SLEDAI of each patient

To determine if the anti-ORF1p autoantibodies present in SLE patients [4, 5] are matched by the presence of this protein in any of their immune cells, we stained polymorphonuclear (PMN) and peripheral blood mononuclear leukocytes (PBMC) from SLE patients or healthy donors with the anti-ORF1p monoclonal antibody 4H1 and counter-stained them with lineage markers. To prevent non-specific staining, 4H1 was directly conjugated to fluorophore (as were the lineage markers) and all steps of the staining protocol included an unlabeled Fc receptor-blocking antibody and 1% normal mouse serum (all antibodies were mouse antibodies). These precautions will eliminate any binding of labeled antibodies through their Fc portions to surface Fc receptors, which are present on neutrophils. Flow cytometry was performed with all combinations, as well as combinations lacking each individual antibody. As shown in Fig. 1A with a representative patient, 35% of the neutrophils, as identified with the lineage marker CD66b (CEACM8), in the PMN fraction were positive for ORF1p, as were 45% of the neutrophils stained by CD14, which is also a present on most neutrophils. In the PBMC fraction, less than 1% of monocytes or B cells were positive in the shown patient (Fig. 1B).

In a cohort of patients (*n* = 13) and healthy donors (*n* = 5), 10 patients had ORF1p^+^ CD66b^+^ neutrophils with the percentage of positive cells ranging from 3–72% with an average of 20.3% (± 26.9%; *n* = 13) (Fig. 1C). The CD66b^+^ cells in the PBMC fraction, which represent low-density granulocytes (based on their neutrophil marker CD66b and presence in the lower density PBMC fraction) potentially with some contaminating regular granulocytes, also varied from 0–82%. The average was 19.5% (± 25.3%, *n* = 10). CD14^+^ monocytes in the PBMC were positive in 2 patients, CD19^+^ B cells in 3 patients, and CD3^+^ T cells in one (Fig. 1C), while these lineages were all negative in heathy donors (Fig. 1D). We noted that the patients with active disease (SLEDAI ≥ 6) had higher percentages of ORF1p^+^ neutrophils (average 28.6%) than those with low disease activity (SLEDAI ≤ 4) (average 1.7%) at the time of the blood draw (*p* = 0.011) (Fig. 1E). This was also true for the low-density granulocytes (*p* = 0.038) with averages of 29.3% vs 4.8% (Fig. 1E), while the trend towards a similar difference in B cells (3.8 vs 0.3%) was not statistically significant. A linear regression analysis of this relationship is also shown (Fig. 1F).

### Identification of L1Hs loci that are transcriptionally active

**Fig. 2:**
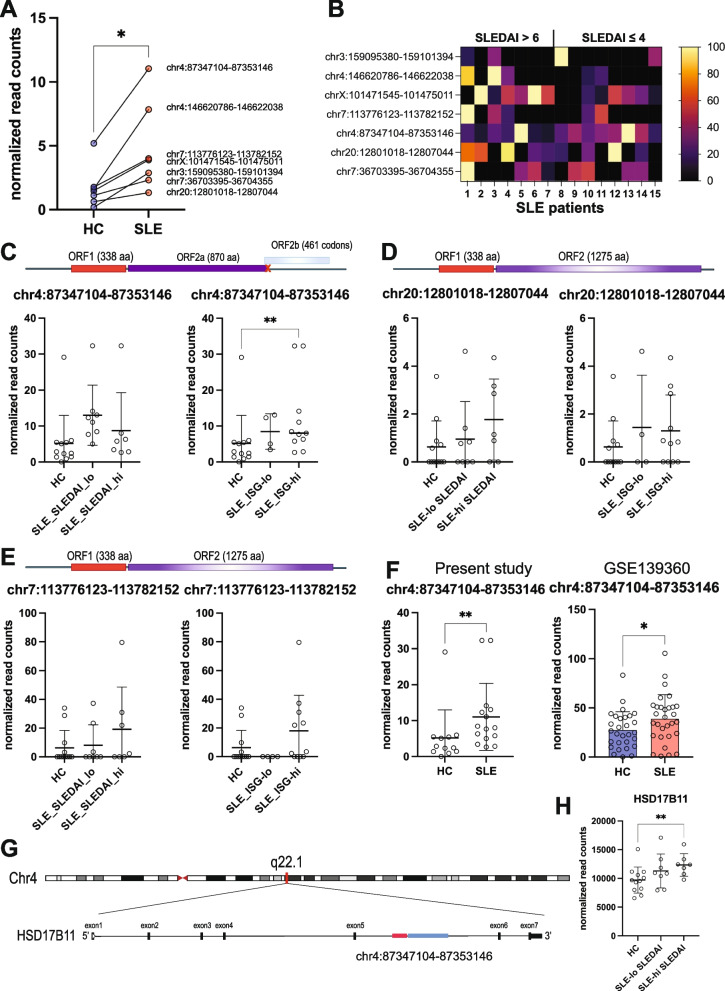
L1Hs expressed at elevated levels in SLE neutrophils. **A** Average normalized read counts of the transcripts uniquely mapping to full-length L1Hs loci that are increased in neutrophils from SLE patients (*n* = 15) and healthy donors (HC; *n* = 12). The loci are identified by their chromosomal location and genomic coordinates. **B**. Heat map representation of individual patient patterns of L1Hs expression. The normalized read counts were adjusted to 100 for the highest expression of each locus. **C** The predicted translation products of the indicated L1Hs element on chromosome 4 and the normalized read counts for it in the healthy controls (HC) in the SLE patients segregated by SLEDAI score into low versus high disease activity (left panel) or by low versus high ISG levels (right panel). Red letters ‘x’ denote premature stop codons, red boxes represent *ORF1* translatable into full-length 338-amino acid residue ORF1p. Purple boxes represent ORF2p; full-length is 1275aa. Note that *ORF2* in this locus has a frame-shift mutation followed by a stop codon that terminates the protein after 870aa residues, which includes intact endonuclease (EN) and reverse transcriptase (RT) domains, but lacks the C-terminal Cys-rich domain (CR), the sequence of which can be found in a different reading frame (pale blue box) and therefore not translated. **D** same data for the indicated L1Hs on chromosome 20, which is full-length and has intact ORF1 and ORF2. **E** Same data for the indicated L1Hs on chromosome 7. **F** Side-by-side data from the present study (left panel) and similarly retrieved data from data set GSE139360 (*n* = 30 SLE, *n* = 29 HC) for the same L1Hs as in panel C. **G** Schematic illustration of the location of the same L1Hs (chr4:87347104–87353146) within the 5^th^ intron of the *HSD17B11* gene. The L1Hs element must be independently transcribed from its 5’ UTR to be poly-adenylated and translated. **H** Normalized read counts for the processed mRNA of HSD17B11 in HC and SLE patients segregated by disease activity. Bars indicate mean ± standard deviation. * denotes *p* values < 0.05, ** *p* < 0.01, and *** *p* < 0.005

To characterize their potential to produce ORF1p, we analyzed L1Hs transcripts in neutrophils from fifteen SLE patients and twelve healthy controls using RNA-seq. This approach has well-known issues with multimapping (*i.e*.,150-bp reads may map to more than one genomic locus) – we therefore focused our analyses on L1 sequences that could be uniquely mapped to a single locus (‘uniquely mapping’). Of these, transcripts from a total of 36 L1Hs loci were present in the patients and healthy controls combined. This number increased to 71 when multi-mapping reads were included, albeit most of them below our cut-off of 10 read counts at least in one sample. Of the uniquely mapping L1Hs transcripts, 7 were elevated > twofold in SLE neutrophils compared to healthy donor neutrophils (Fig. 2A, Supplemental Fig. S1 and Supplemental Table 1). This number was 12 when reads matching more than one locus were included (Supplemental Fig. S2). Interestingly, each of the SLE patients had a unique pattern of transcripts from these L1Hs loci with a tendency of those with active disease (SLEDAI > 6) to express more loci (Fig. 2B). Of the increased 7 uniquely mapping L1Hs, only 3 originate from full-length (> 6,010 bp) L1Hs loci with a 338-codon *ORF1* without stop codons and therefore potentially able to produce ORF1p (Fig. 2A-E). Segregating the SLE patients by SLEDAI score into active versus inactive disease, or by ISG expression into those with elevated (> 2 SD of healthy donors) versus low ISGs, revealed that L1Hs expression tended to be higher both in active disease and in those patients with elevated ISGs (Fig. 2C, D, E), although this was statistically significant only for the L1Hs on chr4:87,347,104–87,353,146 (Fig. 2C). This was also true for the other 4 elevated L1Hs loci (lacking intact ORF1) (Supplemental Fig. S1). We conclude that the ORF1p that we detect in SLE neutrophils is likely the product of 3 distinct L1Hs loci, the individual contribution of which appear to vary between patients.

To expand the analysis, we downloaded a publicly available neutrophil RNA-seq dataset (Gene Expression Omnibus database, accession number GSE139360) from SLE patients (*n* = 30) and healthy controls (*n* = 29). This data set also revealed that L1Hs_chr4:87,347,104–87,353,146 was increased (*p* < 0.05) (Fig. 2F), as was L1Hs_chr3:159,095,380–159,101,394, though the latter was not statistically significant not shown).

Regarding the second L1-encoded protein, ORF2p, which is a reverse transcriptase, our data reveal that only three of the transcribed loci have an intact *ORF1* allowing for translation of *ORF2*, but only two of them have these have an intact *ORF2*, namely L1Hs_chr20:12,801,018–12,807,044 (Fig. 2 D) and L1Hs_chr7:113,776,123–113,782,152 (Fig. 2E), while the other 4 transcripts (Supplemental Fig. S1A-D) have stop codons in *ORF2*, lack portions of *ORF2*, or have stop codons in *ORF1* that halt translation before the brief gap that allows ORF2p to be translated from the bicistronic L1 transcript [42]. Interestingly, L1Hs_chr4:87,347,104–87,353,146 has a frame-shift mutation in *ORF2* predicted to result in an 870-residue protein with a short novel C-terminus, rather than the full-length 1275-residue ORF2p. This truncated ORF2p contains the entire reverse-transcriptase domain but lacks the cysteine-rich C-terminal region, which is required for retrotransposition [43], but not required for reverse-transcriptase activity. We speculate that this truncated ORF2p is an active reverse transcriptase, but does not support L1 retrotransposition: it may remain in the cytosol where it can produce interferonogenic DNA products.

Of potential relevance, L1Hs_chr4:87,347,104–87,353,146 maps to cytogenetic band 4q22.1 and is located in the 5^th^ intron of a gene involved in estradiol metabolism, hydroxysteroid 17-beta-dehydrogenase 11 (*HSD17B11*) on the same negative DNA strand (Fig. 2G). Our RNA-seq data showed that *HSD17B11* is expressed in heathy neutrophils and is increased in SLE neutrophils, particularly from patients with active disease (Fig. 2H). Importantly, transcription of the entire *HSD17B11* will not result in translatable L1Hs RNA since the excision of intron 5 during RNA splicing will not result in nuclear export of an L1-containing RNA with a poly-A tail.

### Expression of epigenetic and transcriptional regulators of L1

Next, we analyzed the expression of regulators of L1 transcription, RNA decay, and other factors influencing L1 biology. Because ORF1p was present predominantly in neutrophils from SLE patients with active disease, we focused our analysis on the samples from patients with active disease (*n* = 7) versus those with inactive disease (*n* = 8), as well as the healthy donors (*n* = 12). We also segregated all the patients by their expression of interferon-inducible genes (ISGs) into those with low (*n* = 4) or high ISGs (*n* = 11).

**Fig. 3:**
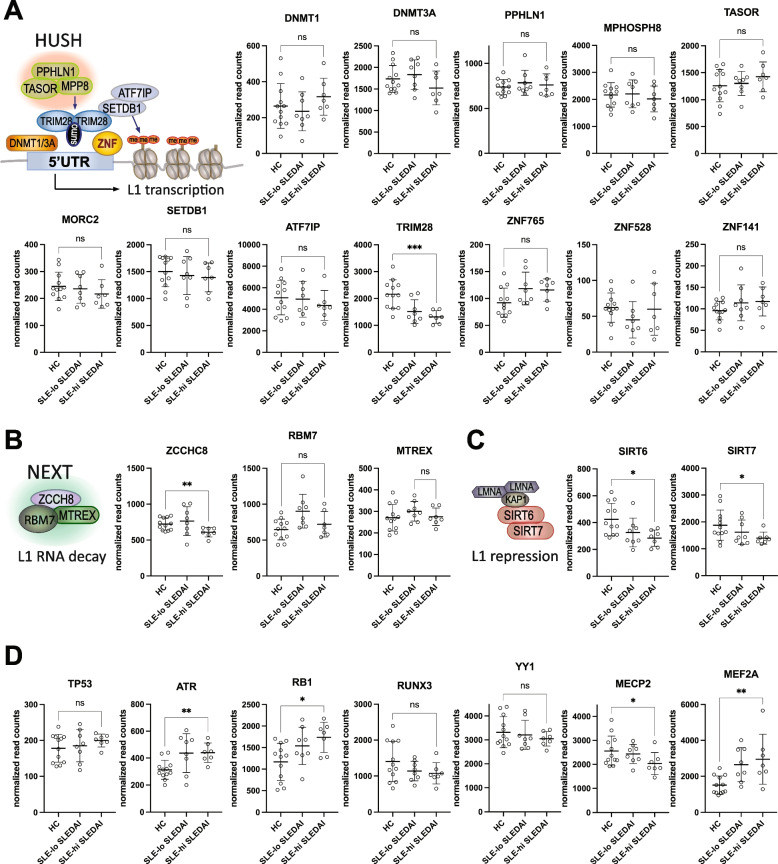
Expression of genes involved in epigenetic, transcriptional, or post-transcriptional regulation of L1 in neutrophils from SLE patients and healthy volunteers. **A** Schematic representation of the HUSH complex and the proteins involved in silencing L1 loci and expression of the indicated genes in HC and SLE patients segregated by disease activity. **B** Schematic representation of the NEXT complex that regulates L1 transcript decay and expression of the indicated genes in HC and SLE patients. **C** Schematic representation of the sirtuins that maintain inactive heterochromatin structure and anchor L1 loci to lamin A in the inner nuclear envelope and expression of *SIRT6* and *SIRT7* in HC and SLE patient neutrophils. **D** Other known regulators of L1 transcription and the expression these genes in HC and SLE neutrophils. Bars indicate mean ± standard deviation. * denotes *p* values < 0.05, ** *p* < 0.01, and *** *p* < 0.005

L1 transcription is effectively silenced by DNA methylation catalyzed by DNA methyltransferase 1 (*DNMT1*) and 3A (*DNMT3A*), the expression of which was not significantly altered in SLE neutrophils (Fig. 3A). DNA methylation, in turn, allows for the binding of the Human Silencing Hub (HUSH) complex [44–46], which consists of the proteins encoded by the genes *MPHOSPH8*, *TASOR*, and *PPHLN1*, in conjunction with *SETDB1*, *ATF7IP*, *TRIM28*, the chromatin regulator *MORC2*, and KRAB-domain containing Zinc-finger factors, such as *ZNF765, ZNF528*, and *ZNF141* (for L1Hs). Transcripts for these genes were unchanged in SLE neutrophils compared to neutrophils from healthy donors, except for *TRIM28* which was decreased in a statistically significant manner (*p* < 0.005) (Fig. 3A). These data do not reveal any global reduction in these mechanisms of L1 repression, but do not preclude the possible existence of locus-specific alterations.

L1 transcripts are also subject to decay through the action of the nuclear exosome targeting (NEXT[47]) complex (Fig. 3B), which interacts with the HUSH complex. The genes constituting this complex are *ZCCH8*, *MTREX4*, and *RBM7*, of which *ZCCH8* was decreased in neutrophils from active SLE compared to neutrophils from inactive SLE or healthy controls (Fig. 3B). The other genes of the NEXT complex were unchanged. L1 loci are repressed in many cells, in part, by the sirtuins SIRT6[48] and SIRT7[49], which inactivate these loci via the maintenance of repressive heterochromatin anchored to lamin A in the inner nuclear envelope. Interestingly, expression of both *SIRT6* and *SIRT7* were decreased in SLE neutrophils, particularly from those with active disease (Fig. 3C). This may contribute to increased transcription of L1Hs in SLE.

Additional transcription factors or epigenetic modulators reported to regulate L1 expression, were elevated (*ATR*, *RB1*, *MEF2A*) or downregulated (*MECP2*) moderately, reaching statistical significance (Fig. 3D). It is not clear at this time how these alterations would affect L1Hs transcript levels in SLE.

### Expression of regulators of L1 biology

**Fig. 4:**
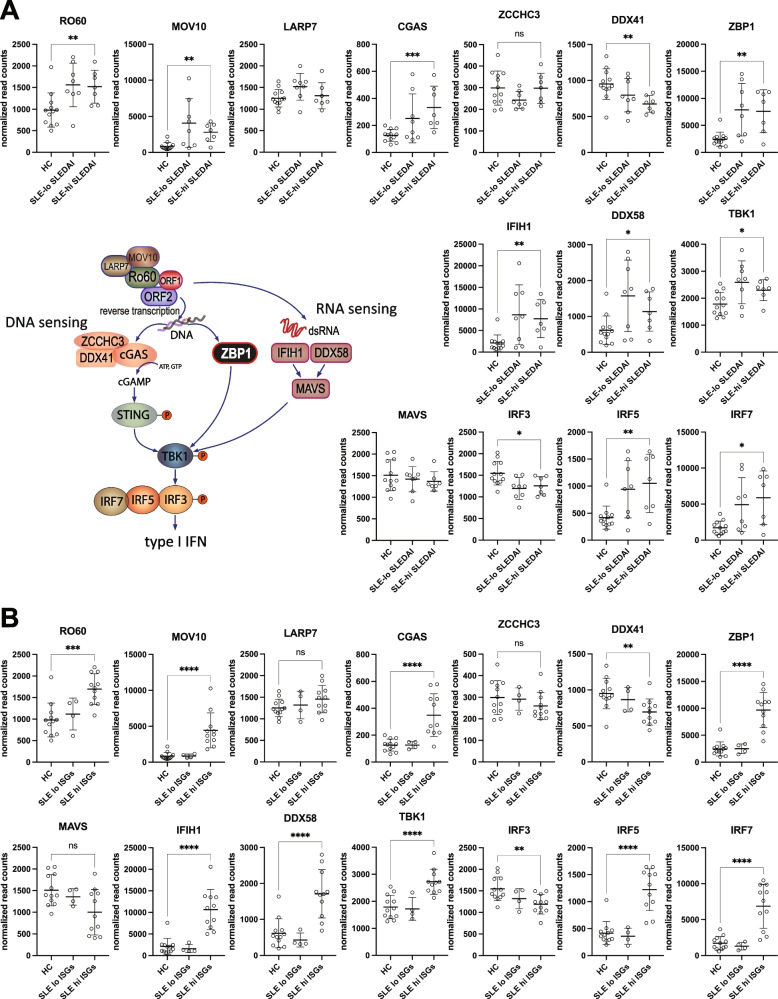
Expression of genes involved in post-translational regulation of ORF1p and ORF2p or nucleic acid sensing and signaling to type I interferon induction in neutrophils from SLE patients and healthy volunteers. **A** Schematic representation of the molecular machinery upstream and downstream of the L1 proteins and normalized read counts of the indicated genes in HC and SLE patients segregated by disease activity. **B** Normalized read counts of the same genes in HC and SLE patients segregated in interferon gene signature (ISGs). Bars indicate mean ± standard deviation. * denotes *p* values < 0.05, ** *p* < 0.01, *** *p* < 0.005, and *** *p* < 0.001. ns, not significant

The L1-encoded ORF1p and ORF2p proteins exist in cells as macromolecular assemblies with several other RNA-binding proteins, at least some of which influence their activity. Transcripts of *RO60* and *MOV10* were strongly upregulated in SLE neutrophils (*p* < 0.001), while *LARP7* was unchanged (Fig. 4A). This effect was evident in neutrophils from both patients with inactive and active disease (Fig. 4A), but was much more clearly influenced by type I interferons (Fig. 4B).

### SLE neutrophils have upregulated nucleic acid-sensors

The expression of many of the genes that encode nucleic acid sensors and components of their signaling to type I interferon production (Fig. 4A) were significantly altered in SLE neutrophils compared to healthy controls: transcripts for the DNA sensors *CGAS* and *ZBP1* and the two RNA sensors RIG-1 (*DDX58*) and MDA5 (*IFIH1*) were significantly increased, as were the transcripts for TBK1 (Fig. 4A), while *DDX41* was downregulated (Fig. 4A). Interestingly, the IRF3 transcription factor was significantly downregulated, while the related IRF5 and IRF7 instead were increased (Fig. 4A).

Because patients with a high type I interferon signature appear to have a molecularly distinct form of SLE, we segregated the patients by interferon signature (i.e. ISG levels > 95 percentile of the healthy control distribution), which revealed a much more pronounced upregulation of the genes that encode nucleic acid sensors and components of their signaling pathway in the ISG-high patients (Fig. 4B). Collectively, these data illustrate an enhanced capacity to recognize pathogenic nucleic acids and drive type I interferon production.

### Neutrophils contain reverse transcriptase inhibition-sensitive interferon stimulated genes (ISGs) and IFNβ

**Fig. 5:**
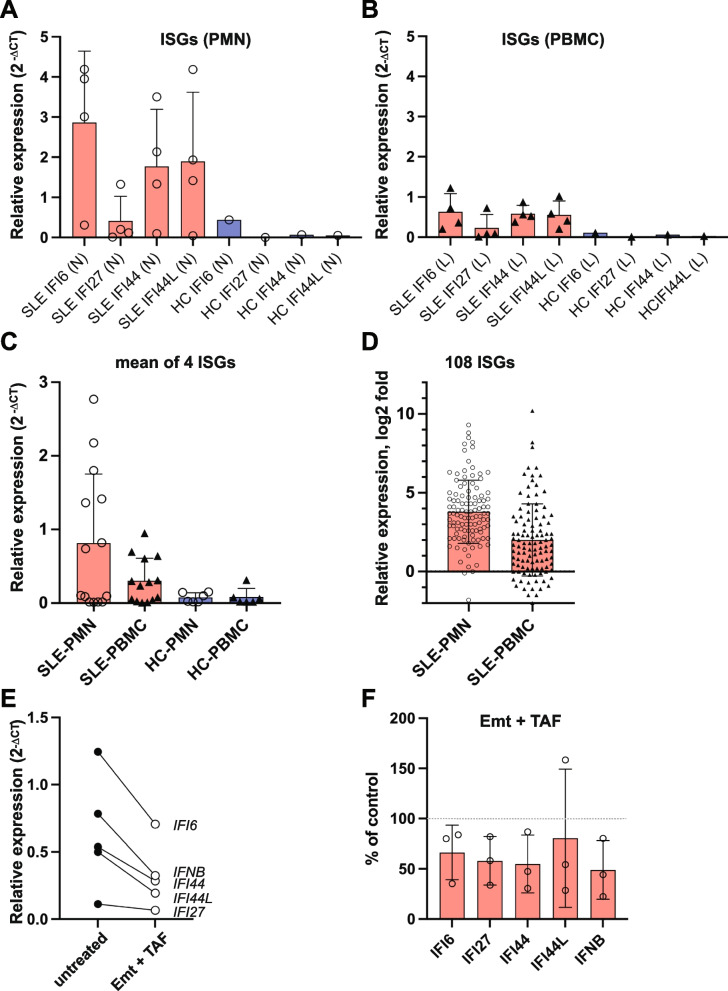
RT-sensitive interferon-inducible genes and *IFNB* in neutrophils and lymphocytes from SLE patients and healthy volunteers. **A** Real-time PCR quantitation of four representative interferon-stimulated genes (ISGs) from neutrophils from SLE patients (*n* = 4) or HC (*n* = 1). The data represent expression normalized by GAPDH signal. **B** Real-time PCR quantitation of the same ISGs from mononuclear cells from SLE patients (*n* = 4) or HC (*n* = 1). The data represent expression normalized by GAPDH signal. **C** Composite scores (mean) of the same ISGs from neutrophils from SLE patients (*n* = 14) or HC (*n* = 7). **D** Increased expression of one hundred and eight ISGs identified by RNA-Seq in SLE neutrophils (SLE-PMN) relative to healthy donor neutrophils and in SLE mononuclear cells (SLE-PBMC) relative to healthy donor mononuclear cells. The data points represent the average of each gene among the patients relative to the average expression of the same gene in healthy controls. **E** Real-time PCR quantitation of the ISGs and *IFNB* from neutrophils kept in cell culture for 4 h without or with 10 µM emtricitabine and 1.25 µM tenofovir alafenamide. The data represent the average of 3 independent experiments. **F** The same data as in E shown as individual patients (*n* = 3) and as percent of control (100% = without inhibitors)

To begin to dissect the potential consequences of the expression of intact and functional L1Hs loci in SLE neutrophils, we first measured by real-time PCR a commonly used set of type I interferon-inducible genes (*IFI6, IFI27, IFI44,* and *IFI44L*) in both neutrophils and lymphocytes from SLE patients and heathy volunteers. As expected, some SLE patients expressed these genes at elevated levels, and others at levels comparable to healthy donors (Fig. 5A). Compared to the housekeeping gene *GAPDH*, those SLE patients with elevated expression (often referred to as ‘interferon signature positive’), had 2–fourfold higher expression of these genes in their neutrophils (Fig. 5A) compared to their lymphocytes (Fig. 5B). This is also evident when the average value of the 4 ISGs are calculated from each patient (Fig. 5C). Similar results were seen in our RNA-seq data (Fig. 5D): 108 ISGs were increased up to 445-fold in SLE neutrophils compared to healthy donor neutrophils, while these transcripts were 2- to 6.4-fold lower (*p* = 0.0045) in the lymphocytes from the same donors.

A 4-h treatment of SLE neutrophils from three independent donors with the combination of the two reverse transcriptase inhibitors, emtricitabine and tenofovir alafenamide, which are known to be effective against ORF2p [50, 51], resulted in a decline in the ISGs, as well as reduced *IFNB* expression, compared to neutrophils treated for the same time with medium alone (Fig. 5E and F). Cell viability remained high as assessed by Trypan Blue exclusion and expression of the housekeeping gene GAPDH were identical between these samples (panel C in Supplementary Figure S2). We cannot of course exclude the possibility that emtricitabine and tenofovir influenced *IFNB* expression by non-specific effects.

## Discussion

Our data show that SLE patients with active disease exhibit elevated levels of L1Hs transcripts and ORF1p protein predominantly in their neutrophils. The set of L1Hs loci that are expressed in patient neutrophils differs somewhat between individual patients. In addition, neutrophils from SLE patients as well as from healthy donors contain transcripts from additional L1Hs loci, which are truncated and/or unable to produce ORF1p or ORF2p. Few of these are differentially expressed. There are also transcripts from numerous older L1 families, exceedingly few of which encode intact L1 ORF proteins (and none of which are known to be functional). Our finding that *TRIM28* of the HUSH complex [44–46] is reduced in SLE neutrophils compared to neutrophils from healthy donors could potentially contribute to L1Hs expression. Reduced expression of *SIRT6* and *SIRT7* could also contribute to the higher levels of L1Hs transcripts in SLE, although it is not at this time known if the reduced mRNAs result in reduced levels of SIRT6 and SIRT7 proteins. Polymorphisms in *DNMT1* are also associated with SLE [52] and the gene is reportedly expressed at lower levels in SLE immune cells than in healthy controls [53]. Reduced genomic methylation can also be caused by certain drugs, such as hydralazine and procainamide, which are known to induce SLE (so-called ‘drug-induced lupus’)[54]. Another well-known trigger of lupus flares, ultraviolet-B light [55], also induces genomic hypomethylation [56]. Our identification of the active L1Hs loci in SLE neutrophils will enable a deeper analysis of their epigenomic landscape, including transcription factors associated with their 5’-untranslated regulatory regions. Furthermore, it should also be stated that detectable ORF1p in SLE neutrophils does not necessarily depend solely on transcription of full-length, intact L1Hs loci. There are reports of translational control of L1 transcripts, for example through its CpG-rich 5’-UTR [57] or microRNAs [58]. Hence, the increased presence of ORF1p in SLE neutrophils could, at least in part, be due to dysregulated translation of L1Hs transcripts or stability of the ORF1p protein.

Another unexpected finding was that the highest abundance, potentially functional L1Hs transcript in SLE neutrophils contained a frame-shift and premature stop codon in *ORF2*. If translated, this would produce an 870-amino acid residue protein with an intact reverse transcriptase domain but lacking the cysteine-rich C-terminal domain; the missing domain is required for retrotransposition, but not for reverse transcriptase activity[50]. We speculate that the truncated ORF2p encoded by the L1Hs at 4q22.1 remains in the cytosol, where it would be well poised to synthesize DNA that triggers cytosolic DNA sensors.

An important finding in our study was that neutrophils from SLE patients also contain higher levels of IFN-inducible gene transcripts than lymphocytes from the same patients. While this could be due to type I IFNs present in the circulation of the patients, the expression of these genes persisted and even increased in the isolated and washed neutrophils over 4 h in culture. Inclusion of the two RTIs, emtricitabine and tenofovir alafenamide, both of which have been shown to inhibit the activity of ORF2p [50, 51], reduced the expression of these IFN-inducible genes. Neutrophils also contained the mRNA for IFNβ, which also declined by approximately half in the presence of these drugs. Unfortunately, neutrophil viability in vitro does not allow for longer incubations. Nevertheless, it seems that cell-intrinsic type I IFN likely contributed to the induction of IFN-response genes and that this cell-intrinsic IFN was dependent on ongoing reverse transcription. L1 ORF2p is also the reverse transcriptase responsible for the aberrant DNA [15, 59] that drives type I IFN production and disease in patients with the interferonopathy Aicardi-Goutières syndrome [60]. In a small clinical trial in these patients, reverse transcriptase inhibition flat-lined the IFN-inducible gene signature for the duration of drug dosing [61].

## Conclusions

Our data support a contribution of dysregulated L1Hs expression, translation of L1Hs transcripts, and the biological action of ORF1p and ORF2p to SLE disease. The correlation of SLE disease activity with ORF1p quantity in neutrophils and the presence of reverse transcriptase-sensitive IFN-inducible gene transcripts and IFNβ in these same cells are compatible with a connection between L1 biology and SLE pathogenesis. Ultimately, the importance of L1 dysregulation in SLE pathogenesis will need to be evaluated in a double-blinded placebo-controlled clinical trial with potent and selective reverse transcriptase inhibitors.

## Methods

### SLE patients

Freshly drawn blood was obtained from adult patients with SLE and healthy age-matched individuals recruited through the University of Washington, Division of Rheumatology Biorepository to participate in research studies at the University of Washington Medical Center, Seattle, WA. The study was approved by the institutional ethics board (STUDY00006196) and informed written consent was obtained from all participants according to the Declaration of Helsinki.

### Isolation of neutrophils and peripheral blood mononuclear cells

Polymorphonuclear (PMN) and peripheral blood mononuclear cells (PBMC) were isolated simultaneously from freshly drawn venous blood by gradient centrifugation on PolymorphPrep according to the manufacturer’s instructions. The PMN fraction was > 95% CD66b^+^ and > 85% CD15^+^. The majority of PBMC were CD3 + T cells, plus 5–15% CD19^+^ B cells, 2–6% CD14^+^ monocytes, and 3–10% CD66b^+^ low-density granulocytes. The cells were washed and suspended in Hanks’ buffered salt solution at a range of 1–10 × 10^6^ cells/ml before use in various experiments.

### Flow cytometry

Cells were stained with anti-CD66 (eBioscience), anti-CD14 (BioLegend), and anti-CD19 (Biolegend) antibodies covalently labeled with PE-Cyanine7, PerCP-Cyanine5.5, and APC-Cyanine7, respectively. Following fixation with 1% paraformaldehyde in phosphate-buffered saline and permeabilization with saponin, cells were stained with an anti-ORF1p antibody 4H1 [27] (MilliporeSigma) covalently labeled with Cy5. An unlabeled blocking antibody to FcγRIIA (CD32A) (Sino Biological Inc.) was also added at this step. After a 1 h incubation, the cells were washed and resuspended in phosphate-buffered saline with saponin and added to a 96-well plate at 1 million cells in 100 µl per well. All steps were performed in the presence of 1% mouse serum to block non-specific binding. Flow cytometry was run on a CytoFLEX Flow Cytometer (Beckman Coulter) and data analysis was done with FlowJo (Becton, Dickinson and Company). FMO (fluorescence minus one) wells were used to determine the cut-off point between background fluorescence and positive populations. Compensation runs were also performed to address any potential overlap of fluorophore emission. Such runs were done with UltraComp eBeads Plus compensation beads (ThermoFisher Scientific).

### Treatment of neutrophils with reverse transcriptase inhibitors

Cells were resuspended at 5 × 10^6^ cells in 1 ml of RPMI media containing 10% fetal bovine serum and 1 × Penicillin–Streptomycin in 12-well cell culture plates. Two reverse transcriptase inhibitors (RTIs), emtricitabine (Emt) and tenofovir alafenamide (TAF), were added to the experimental cultures at a final concentration of 10 µM and 1.25 µM, respectively. Note that these are at a ratio of 1:8 based on their therapeutic dose ratio in the combination drug, Descovy® (Emt 200 mg/TAF 25 mg), which is FDA approved for the treatment of HIV infection. The experimental (treated) and control (untreated) cell cultures were then incubated at 37 °C with 5% CO_2_ for 4 h before harvesting for RNA extraction.

### RNA isolation

Extraction of RNA was performed by using a hybrid protocol of Trizol Reagent (Invitrogen cat# 15,596,026) and RNeasy Micro Kit (Qiagen cat. no. 74004). Cells were lysed and homogenized with Trizol Reagent followed by addition of chloroform to separate DNA and proteins from the RNA to which β-mercaptoethanol was added for additional RNase inhibition. The RNA was precipitated with 70% ethanol, passed through a RNeasy MinElute spin column, and treated with DNase I on the column. The spin column was subsequently washed extensively with the kit-supplied wash buffers, followed by additional conditioning with 70% ethanol before eluting the RNA with RNase-free water.

### Real-time quantitative polymerase chain reaction (PCR)

A two-step reverse transcriptase (RT)-quantitative PCR method was utilized by first performing cDNA synthesis using Qiagen QuantiTect Reverse Transcription kit with integrated removal of genomic DNA contamination (Qiagen cat# 205,313) and ran on Applied Biosystems PCR Thermal Cycler. The resultant cDNA was subsequently used as a template for the qPCR setup using the Qiagen QuantiTect SYBR Green PCR kit (Qiagen cat# 204,143) and ran on Applied Biosystems StepOne Plus Real-Time PCR Thermal Cycler. The qPCR incorporated ROX dye as a passive reference to normalize for minor variations in fluorescent intensity between reactions, and GAPDH was used as the housekeeping gene. The relative expressions of L1, *HSD17B11*, and IFN inducible genes were calculated using the 2^−∆CT’^ method (for non-drug-treated samples) and the 2^−∆∆CT^ method (for drug-treated samples) with normalizations against GAPDH. The following primers were used: *L1* forward 5’-ACCAAAAGTAGATAAAACCACAAAGA-3’, reverse 5’-GAACTGCGTTCCTTTGGAGG-3’ (amplicon 102 bp); *HSD17B11* forward 5’-ACATGTCTGTGTCCTAATTTCGT-3’, reverse 5’-TCCCATGCATCAGCCTGTTT-3’ (amplicon 109 bp); *IFNB* forward 5’-ACGCCGCATTGACCATCTAT-3’, reverse 5’-GTCTCATTCCAGCCAGTGCT-3’ (amplicon 85 bp). Primer sequences from Ward et al. [62]: *IFI6* forward 5’-TGCTACCTGCTGCTCTTCA-3’, reverse 5’-TCAGGGCCTTCCAGAACC-3’ (amplicon 97 bp), *IFI27* forward 5’-TTGTGGCTACTCTGCAGTCA-3’, reverse 5’-CCCAGGATGAACTTGGTCAA-3’ (amplicon 64 bp); *IFI44* forward 5’-GGCTTTGGTGGGCACTAATA-3’, reverse 5’-TGCCATCTTTCCCGTCTCTA-3’ (amplicon 77 bp); *IFI44L* forward 5’-GCAAAAGTGAAGCAAGTTCACA-3’**,** reverse 5’-GAACCTCACTGCAATCATCCA-3’ (amplicon 91 bp).

### RNA sequencing and bioinformatics analysis of L1Hs and epigenetic factors

RNA concentration and purity were determined by checking their spectrophotometric absorbances at A260/A280 using BioTek Take3 microplate reader. RNA integrity was verified by performing nondenaturing gel electrophoresis on 1% agarose gel to confirm the presence of the 28S and 18S rRNAs. RNA library preparation was performed using oligo dT selection. Paired-end strand-specific RNA sequencing libraries were generated. Sequences were aligned to the hg38 genome with the STAR aligner [63] TElocal which is part of the TEtranscripts [64] package was used to estimate loci-specific LINE-1 read counts, first time counting only uniquely mapped elements and a second time allowing multimappers. Since younger families of L1HS have accumulated less mutations than older ones, they are more likely to be very similar to each other resulting in multimapping. The DNA sequence for the LINE-1 locus at 4q22.1 (chr4:87,347,104–87,353,146) as annotated by the RepeatMasker database (Smit AFA, Hubley R, and Green P. RepeatMasker Open-3.0. http://www.repeatmasker.org. 1996–2010) was obtained from the UCSC browser [65]. DESeq2 [66] was used to normalize counts and to perform differential gene testing. Reported *p* values were adjusted for multiple comparisons. Read counts < 10 were excluded from L1Hs analysis. All RNAseq data will be deposited in GEO.

### Patient demographics for the RNAseq cohort

The SLE patients (*n* = 15) included in the RNA sequencing data set consisted of 13 female and 2 male patients, age 22–74, and white (*n* = 5), hispanic (*n* = 5), native American (*n* = 1), Asian (*n* = 3), or black (*n* = 1). Two had verified lupus nephritis. They had SLEDAI scores 8–19 (*n* = 7) or 0–4 (*n* = 8), denoted as active disease and inactive disease, respectively. As determined by having an average expression of 108 ISGs higher than the 95 percentile of the same genes in healthy controls, patients were divided into ISG-high (*n* = 11) versus ISG-low (*n* = 4). The healthy controls (*n* = 12) were sex (3 males, 9 females) and age (23–63) matched and did not have a rheumatic disease.

### Statistical analysis

The statistical significance of non-parametric data set from patient samples was calculated using the Mann–Whitney U-test. A *p*-value < 0.05 was used as the cut-off for statistical significance; values < 0.05 are denoted with *, < 0.01 with **, < 0.005 with ***, and < 0.001 with ****. GraphPad Prism 9.5.0 was used for all statistical analyses.

## Supplementary Information

**Additional file 1: Supplemental Fig. S1. **Expression of the other 4 L1Hs transcripts that are increased in neutrophils from SLE patients. **A** The predicted lack of translation products from the indicated L1Hs element on chromosome 4 and the normalized read counts for its transcript in the healthy controls (HC) in the SLE patients segregated by SLEDAI score into low versus high disease activity (left panel) or by low versus high ISG levels (right panel). **B** Same for the indicated L1Hs on chromosome X. Note that the L1 transcript is bicistronic: ORF2 can only be translated if ORF1 is translated to its end. **C** Same for the indicated L1Hs on chromosome 3. **D** Same for the indicated L1Hs on chromosome 7. Note that this locus is truncated to only the C-terminus of ORF2, which is not likely translated at all. Pale blue boxes are portions of ORF2 that are not translated; full-length is 1275 codons. Red letters ‘x’ denote premature stop codons. Pink boxes denote truncated ORF1. Domains of ORF2 are the endonuclease (EN), reverse transcriptase (RT), and C-terminal Cys-rich domain (CR).

**Additional file 2: Supplemental Fig. S2. **Expression of the 12 L1Hs transcripts that are increased in neutrophils from SLE patients using the less stringent approach of allowing for reads that map to more than one genomic location (because they are identical over the 150 bp reads). **A** Heat map representation of individual patient patterns of expression of the 12 L1Hs. The normalized read counts were adjusted to 100 for the highest expression of each locus. **B** The normalized read counts for the additional loci (compared to Fig. 2) in the healthy controls (HC) in the SLE patients segregated by SLEDAI score into low versus high disease activity (left panel) or by low versus high ISG levels (right panel). **C** Individual GAPDH house-keeping gene values as C^T^ from the real-time PCR reaction with HC or SLE neutrophils treated with medium alone (no drug) or with 10 µM emtricitabine and 1.25 µM tenofovir alafenamide for 4 h (drug) as in Fig. 5E and F.

**Additional file 3: Supplemental Table 1. **Individual uniquely mapping read counts of the 7 L1 elements expressed at elevated levels in SLE patients.

## Data Availability

All data will be made available to qualified researchers upon request. The data in the Gene Expression Omnibus database can be retrieved through accession number GSE139360.
