# Supplementary figures and images for: Expression of L1 retrotransposons in granulocytes from patients with active systemic lupus erythematosus
Source: Mob DNA. 2023 May 10;14:5. doi: 10.1186/s13100-023-00293-7 (PMC10170740; doi:10.1186/s13100-023-00293-7)

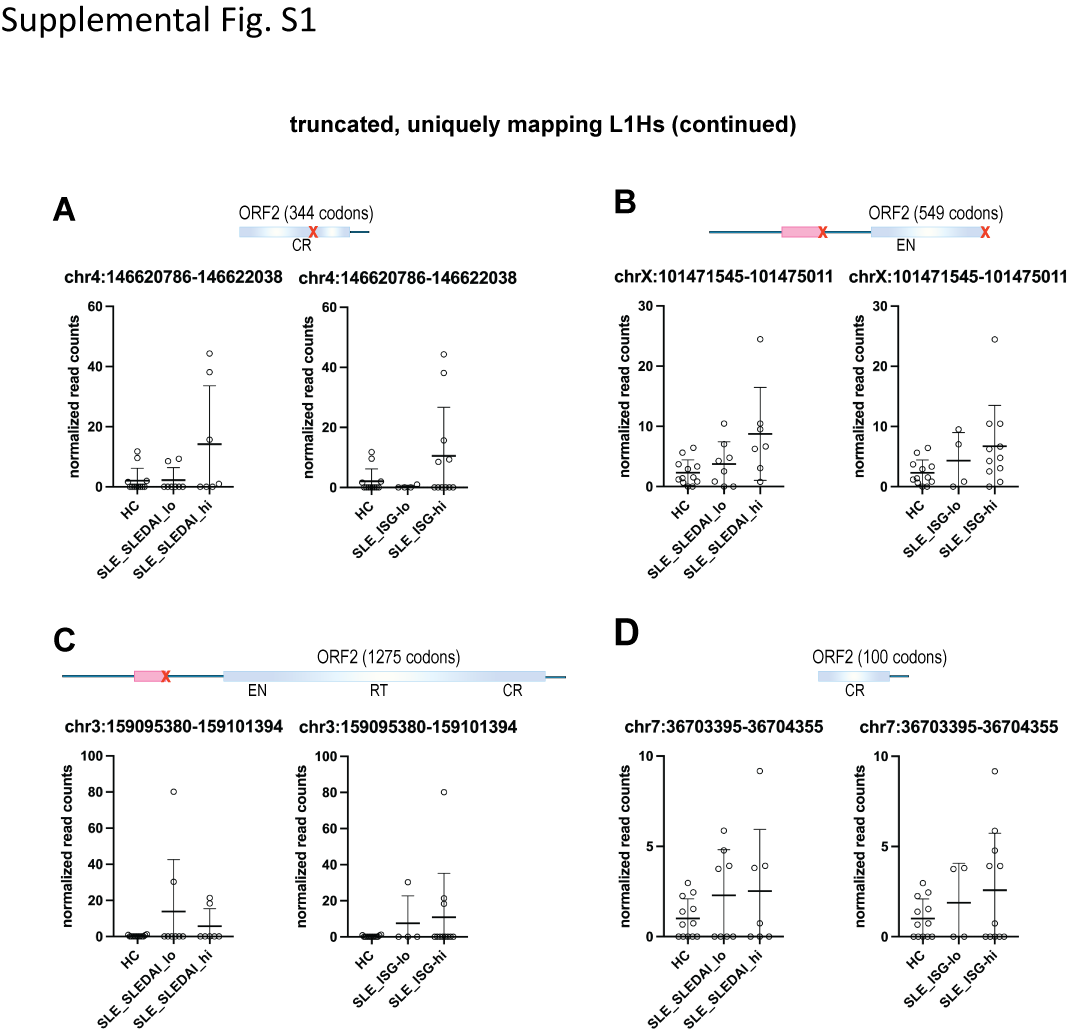

Supplement: Supplementary file 1 — Additional file 1: Supplemental Fig. S1. Expression of the other 4 L1Hs transcripts that are increased in neutrophils from SLE patients. A The predicted lack of translation products from the indicated L1Hs element on chromosome 4 and the normalized read counts for its transcript in the healthy controls (HC) in the SLE patients segregated by SLEDAI score into low versus high disease activity (left panel) or by low versus high ISG levels (right panel). B Same for the indicated L1Hs on chromosome X. Note that the L1 transcript is bicistronic: ORF2 can only be translated if ORF1 is translated to its end. C Same for the indicated L1Hs on chromosome 3. D Same for the indicated L1Hs on chromosome 7. Note that this locus is truncated to only the C-terminus of ORF2, which is not likely translated at all. Pale blue boxes are portions of ORF2 that are not translated; full-length is 1275 codons. Red letters ‘x’ denote premature stop codons. Pink boxes denote truncated ORF1. Domains of ORF2 are the endonuclease (EN), reverse transcriptase (RT), and C-terminal Cys-rich domain (CR). [file 13100_2023_293_MOESM1_ESM.png]

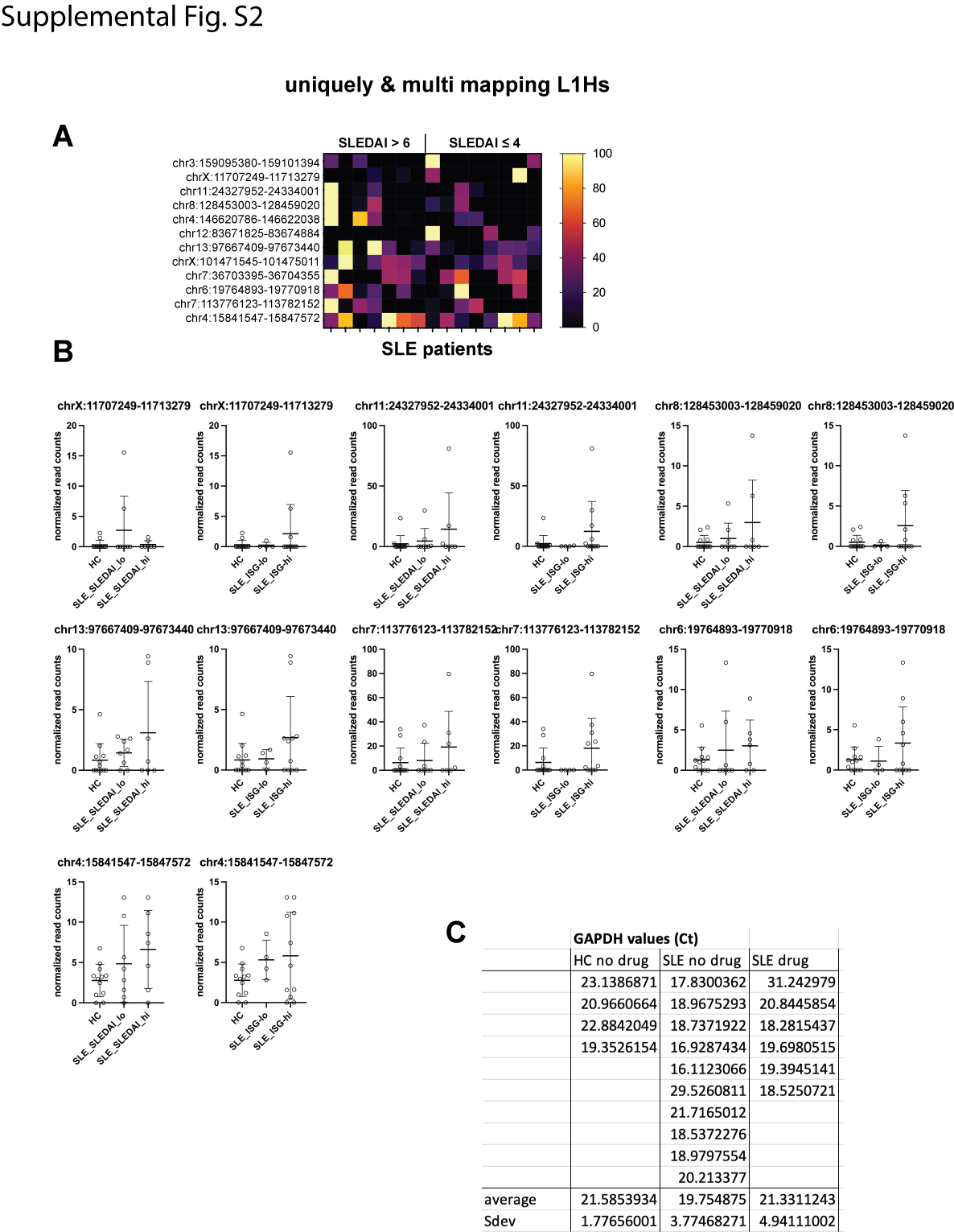

Supplement: Supplementary file 2 — Additional file 2: Supplemental Fig. S2. Expression of the 12 L1Hs transcripts that are increased in neutrophils from SLE patients using the less stringent approach of allowing for reads that map to more than one genomic location (because they are identical over the 150 bp reads). A Heat map representation of individual patient patterns of expression of the 12 L1Hs. The normalized read counts were adjusted to 100 for the highest expression of each locus. B The normalized read counts for the additional loci (compared to Fig. 2) in the healthy controls (HC) in the SLE patients segregated by SLEDAI score into low versus high disease activity (left panel) or by low versus high ISG levels (right panel). C Individual GAPDH house-keeping gene values as CT from the real-time PCR reaction with HC or SLE neutrophils treated with medium alone (no drug) or with 10 µM emtricitabine and 1.25 µM tenofovir alafenamide for 4 h (drug) as in Fig. 5E and F. [file 13100_2023_293_MOESM2_ESM.png]
